# Potentially Inadequate Real-Life Speech Levels by Healthcare Professionals during Communication with Older Inpatients

**DOI:** 10.3390/ijerph20054543

**Published:** 2023-03-03

**Authors:** Anna K. Stuck, Stephan Born, Andreas E. Stuck, Martin Kompis

**Affiliations:** 1 Department of Geriatrics, University of Bern, University Hospital Inselspital Bern, 3010 Bern, Switzerland; 2 Department of ENT, Head and Neck Surgery, University of Bern, University Hospital Inselspital Bern, 3010 Bern, Switzerland

**Keywords:** hearing, geriatrics, elderly, discharge planning meetings, healthcare providers, hospitalized

## Abstract

Background: The aim of this study was to investigate real-life speech levels of health professionals during communication with older inpatients in small group settings. Methods: This is a prospective observational study assessing group interactions between geriatric inpatients and health professionals in a geriatric rehabilitation unit of a tertiary university hospital (Bern, Switzerland). We measured speech levels of health professionals during three typical group interactions (discharge planning meeting (*n* = 21), chair exercise group (*n* = 5), and memory training group *(n* = 5)) with older inpatients. Speech levels were measured using the CESVA LF010 (CESVA instruments s.l.u., Barcelona, Spain). A threshold of <60 dBA was defined as a potentially inadequate speech level. Results: Overall, mean talk time of recorded sessions was 23.2 (standard deviation 8.3) minutes. The mean proportion of talk time with potentially inadequate speech levels was 61.6% (sd 32.0%). The mean proportion of talk time with potentially inadequate speech levels was significantly higher in chair exercise groups (95.1% (sd 4.6%)) compared to discharge planning meetings (54.8% (sd 32.5%), *p* = 0.01) and memory training groups (56.3% (sd 25.4%), *p* = 0.01). Conclusions: Our data show that real-life speech level differs between various types of group settings and suggest potentially inadequate speech levels by healthcare professionals requiring further study.

## 1. Introduction

Hearing impairment is considered a global health issue [1] affecting nearly two thirds of older people aged 70 years and older [2]. Hearing loss has major impacts on underlying morbidity [3,4,5,6,7] and functional impairment resulting in greater disability [8], and is associated with reduced communication and social isolation [4,9,10].

In the hospital setting, hearing impairment can have a profound impact on communication between patients and healthcare providers, especially during conversations related to diagnosis, treatment, and discharge plans. Patient autonomy and decision-making capacity can only be ensured if patients hear and understand what they are told. Even though hearing loss and impairment is a common and well-known issue, a review found that less than one quarter of studies of physician–older patient communication mentioned that hearing loss may affect communication [11].

Prior studies and clinical efforts have primarily focused on interventions directed at improving patients’ hearing (e.g., providing hearing aids) [12,13,14]. Efforts have also been directed at controlling environmental factors. The effect of background noise is well investigated and a maximum threshold of background noise levels has been recommended by the WHO [15] for neonatology settings [16]. A single study by Pearson et al. [17], in the context of evaluating environmental sound levels in schools and hospitals, recorded speech levels produced by nurses talking to each other at the nurses’ station. However, we have not found any studies that measured speech levels of healthcare providers during conversation with older patients. 

There are several different methods to measure speech levels. Even for relatively closely related and well-defined speech audiometry tests, such as the German Freiburger monosyllabic word test and the German OLSA sentence test, two different methods are used: impulse peak levels and the equivalent continuous sound level (L_EQ_), respectively [18]. Although the use of either method results in certain level differences (in this case of up to 5.6 dB), results remain comparable based on the fact that measures of speech levels are ultimately linked to normal hearing thresholds. Another minor issue is the use of different equipment and different ways to calibrate it, but this aspect is negligible [19].

Similarly, room acoustics and the distance between speaker and listener (or microphone) also influence measured speech levels. In small- or medium-sized rooms, such as those used in this study, the critical distance, i.e., the distance at which direct and reverberant sound reach the same level, is almost always below 1 m. At longer distances, the reverberant portion dominates and sound levels are approximately uniformly distributed across the room.

Studies reporting normal levels of natural speech are remarkably rare. Normal speech levels in quiet are known to lie approximately between 60 and 65 dB and to increase, when background noise is present [20]. Speech levels of 65 dB are frequently recommended to represent normal speech in controlled settings, such as in speech audiometry to compare hearing devices [21].

The aim of this study was to evaluate speech levels during real-life communication between health professionals and older patients in a geriatric rehabilitation department. 

## 2. Materials and Methods

### 2.1. Setting 

All recordings were performed in an inpatient geriatric rehabilitation unit in Bern, Switzerland during real-life communication (i.e., no anechoic chamber) between February and March 2019. Patient criteria for admission to the geriatric rehabilitation unit are the following: (1) age > 75 years, (2) direct transfer from acute care hospital, (3) living in the community (i.e., not in a nursing home) prior to acute care hospital admission, (4) potential for functional improvement and discharge home following inpatient rehabilitation. The speech levels of geriatric patients and health professionals were recorded during three typical interactions: (1) discharge planning meeting, (2) memory training group, and (3) chair exercise group. All recorded sessions included a different composition of physicians, therapists, nurses, patients, and relatives. All participating healthcare providers were trained to lead these group interactions with older patients. 

Discharge planning meeting: During this meeting, plans for hospital discharge were discussed with the patient and relatives. Sessions were led by the treating physician, with input from other providers (e.g., therapists and nurses) involved in patient care. These meetings took place in a closed, separate 15 m^2^ carpeted room without relevant background noise. All participants, usually 6–9 individuals, were seated at a round table. The meeting was scheduled for a maximum of 30 min. For most of the speaking time, the physician was providing information to the patient and relatives. Towards the end of the meeting, the patient and relatives were given the opportunity to ask final questions. To record speech levels, we placed the microphone in the center of the round table, which was 1 m from each person attending the meeting. 

Memory training group: The memory training group took place in the same room and was led by a therapist. A group of 5–8 patients and the therapist were seated at a round table. The therapist provided selective instructions to patients on a specific memory task. This group therapy session was scheduled for a maximum of 40 min. To record speech levels, we placed the microphone in the center of the round table, which was 1 m from each person attending the meeting.

Chair exercise group: Sessions took place in a separate small gym (35 m^2^) with 6–9 patients sitting in chairs that formed a circle. The therapist provided instructions on how to perform simple exercises (e.g., instructing patients pass a ball to their neighbor). Between instructions, patients performed the exercises, potentially resulting in a short break in communication. The microphone was placed on a separate chair within the circle (as a substitute for a patient participating in the chair exercise therapy). 

The study was conducted with the approval of the Ethics Committee of the Canton of Bern, Switzerland (Req-2020-00184).

### 2.2. Measurement Device

We used the CESVA LF010 (CESVA instruments s.l.u., Barcelona, Spain) to measure speech levels. The measurement range of the CESVA spans from 50 dBA to 130 dBA. The device only provides anonymous measurements of speech levels (dBA), and does not record conversations or voices. Technical features are provided in detail in the datasheet [22].

### 2.3. Outcome Parameters

The main outcome parameter was mean proportion of effective talk time measured at a potentially inadequate speech level. We chose 60 dBA as the threshold for an adequate speech level based on prior evidence that 60 dBA is the median level of normal conversational speech [17,23,24]. Each minute of speech where 50% of the measured time (L50) was less than 60 dBA was defined a priori as inadequate speech. Secondary outcomes were the proportion of talk time at both low and high extremes, defined by the thresholds of <55 dBA and ≥65 dBA, respectively. Subgroup analyses was performed for discharge planning meetings to compare meetings attended by patients with hearing impairment versus meetings attended by patients with no hearing impairment, and hearing aid versus no hearing aid, respectively. Hearing impairment was measured using the standardized whisper voice test within a distance of 60 cm, defined as incapacity to hear less than 2 out of 3 numbers. 

### 2.4. Analysis

We analyzed the effective talk time within each session. According to the manufacturer’s instructions, the first and last minutes in each session were not analyzed. We also excluded a priori all measured minutes in which the device indicated that L50 was “under range” (“UND”), defined as a speech level that was under the measurement range of the device (50 dBA) for at least 50% of a specific minute. This ensured that conversational pauses were excluded at the beginning and at the end of sessions and while exercises were being performed. 

Descriptive statistics were used to report proportions for categorical data and means and standard deviations for numerical data. Student’s unpaired t-tests were used to compare means between groups. Statistical analyses were performed using MedCalc statistical software (online version, Belgium, 2020). A *p*-value of <0.05 was considered statistically significant. 

## 3. Results

A total of 31 sessions (21 discharge planning meetings, five chair exercise groups and five memory training groups) were recorded and analyzed (Table 1). The mean proportion of talk time with inadequate speech levels (<60 dBA) was 61.6% (standard deviation (sd) 32.0%). 

**Figure 1:**
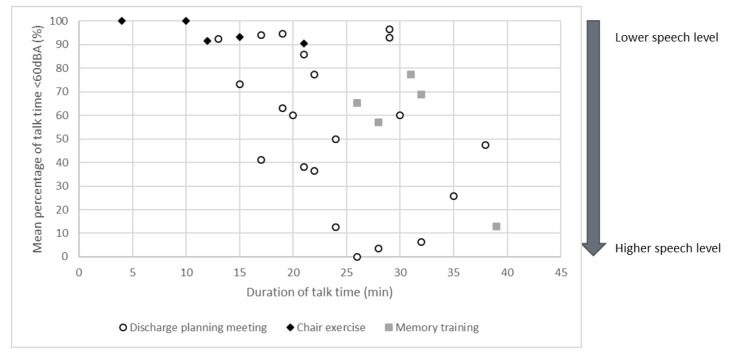
Scatter plot of mean percentage of talk time with potentially inadequate speech level (<60 dBA) per session. Each data point represents an individual session of discharge meeting, chair exercise or memory training group, respectively.

Figure 1 displays the individual data points for each session of discharge planning meetings, chair exercise groups, and memory training groups. The graphic shows that both the duration of talk time and the proportion of talk time with potentially inadequate speech levels differs both between sessions within the same type of setting (e.g., discharge planning meeting) and between the different type of setting (e.g., discharge planning meeting vs. chair exercise group). 

The mean proportion of talk time with potentially inadequate speech level was significantly higher for chair exercise groups compared to discharge planning meetings (mean difference: 40.3%, 95% CI: 9.8 to 70.8%, *p* = 0.01) and memory training groups (mean difference 38.8%, 95% CI: 12.2 to 65.4%, *p* = 0.01). In contrast, there was no statistically significant difference between discharge planning meetings and memory training groups (mean difference: 1.5%, 95% CI: −30.8 to 33.8%, *p* = 0.92).

**Figure 2:**
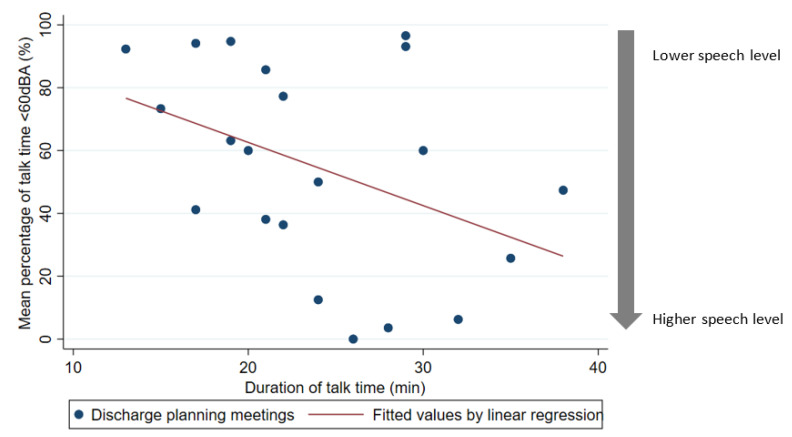
Scatter plot of mean percentage of talk time with potentially inadequate speech level (<60 dBA) per session in discharge planning meetings with fitted regression line. Each data point represents an individual session of discharge planning meeting.

Figure 2 displays discharge planning meetings only including fitted values by linear regression. The regression coefficient between mean percentage of talk time <60 dBA and duration of talk time was −2.03 (95% CI: −4.2 to 0.14; *p* = 0.07). 

In the subanalyses for discharge meetings, mean speech level (LAeq) was 66.0 ± 3.4 dBA in in sessions involving a patient with a hearing device compared to 65.8 ± 2.9 dBA without hearing device, which is statistically non-significant (*p* = 0.90). Similarly, for sessions involving a patient with a hearing impairment versus no hearing impairment means of speech level (LAeq) were statistically not significantly different (65.7 ± 1.9 with hearing impairment vs. 65.9 ± 3.0 dBA without hearing impairment, *p*-value = 0.91).

## 4. Discussion

Our findings reveal that real-life speech levels of healthcare providers during the majority of interactions with older patients in a rehabilitation hospital were lower than what is considered a threshold of what is a speech level at which a patient should be able to acoustically hear what is being said. 

This novel finding is concerning, especially as it relates to discharge planning meetings. The goal of patient-centered care is to provide care that is aligned with a patient’s preferences, values, and goals. Providing patients with information and education about their condition and care options is one of the principles of the patient-centered care model. Our results suggest that the speech level of healthcare providers may be potentially inadequate possibly representing an unrecognized barrier to informed decision making by patients. In the subgroup analysis of the discharge planning meeting, we found a trend for a correlation between a longer duration of talk time and a lower percentage of low speech levels <60 dBa. A possible explanation might be that sessions that were already held at a low speech level included more minutes with speech levels under the range resulting in exclusion of these minutes under the range and resulting in a shorter duration of talk time. However, the fitted regression values were statistically not significant in this subanalysis.

We also found low speech levels in therapy sessions. While the focus of memory training groups is on communication, the focus of a chair exercise group is more on the practical demonstration of a specific exercise and patients can copy the exercise, even if they do not hear the verbal instruction. Nevertheless, effective communication is needed to facilitate patient satisfaction and adherence with the therapy. Similarly, low speech levels in memory training groups might hinder patients from engaging in therapeutic activities and thus negatively affect clinical outcomes. 

We found no other studies that reported absolute values of healthcare providers’ speech levels. However, several studies have looked at the impact of hearing loss on patient understanding and satisfaction. In one study, 22% of hospitalized patients reported that they did not understand when the doctor spoke to them and 52% thought communication with medical staff could be improved [25]. Foss et al. identified “hearing ability” as the only significant factor affecting participation of a patient in a discharge planning meeting [26]. Another qualitative study illustrating patient perspectives taking part in a discharge meeting found that patients felt uncertain, standing outside, and strange [27]. Reed et al. reported that hearing loss was associated with being less satisfied with healthcare [28]. These studies suggest that communication with healthcare providers is an issue for older patients in various settings and supports the validity of further exploration of speech levels as another barrier. 

Clearly, low speech levels and higher hearing thresholds can both decrease speech intelligibility. To give an order of magnitude, decreasing presentation levels by 30 dB or, alternatively, increasing the broadband hearing threshold of a listener by the same amount decreases speech understanding for monosyllabic German words from 100% at 65 dB to approximately 50% [29]. Speech levels are hearing thresholds that are not the only parameters influencing speech intelligibility. Even with the same hearing thresholds, speech understanding tends to deteriorate with age [30]. On the other hand, speaking clearly can increase speech intelligibility even without using higher speech levels [31].

The strengths of our study include the measurement of speech levels during real-life situations using a precise, well-calibrated device and inclusion of a variety of healthcare providers (physicians, therapists, nurses). 

There are several limitations to our study. First, our results are from a single-site geriatric inpatient rehabilitation unit and may not be generalizable to other settings. However, the fact that speech levels were low even among healthcare providers who had been trained to communicate in group settings with older patients suggests that inadequate speech levels might be equally, or even more prevalent, among providers who are not trained in geriatrics. Second, we only investigated group interactions. Communication between providers and older patients in 1:1 settings may show different patterns of speech levels. Third, findings of statistical analyses are explorative, and need to be interpreted with caution due to limited number of observations. Fourth, it could be argued that conversation pauses within a particular minute could have led to the underestimation of the observed speech levels in the present study. However, this effect is unlikely as we ensured analysis of effective talk time with a minimum of 50 dBA. Fifth, the visibility of the recording device during measurement could have caused healthcare providers to artificially speak louder (Hawthorne-effect), thus resulting in an overestimation of speech levels. However, if this occurred, then the proportion of potentially inadequate speech levels may be even greater. We also cannot explain the observed speech levels as being due to an individual person having a particularly stronger or softer voice, because data are reflecting speech levels of various healthcare professionals and settings, respectively. Finally, we did not test for effective hearing and cognitive comprehension (speech intelligibility) of the patients in the group settings. In a future study, both sound levels and comprehension should be systematically evaluated. 

Our observations have several implications, both for future studies and clinical implementation. In line with the “Hospital Elder Life Program” (HELP), hearing needs to be addressed as a key intervention during hospitalization of an older patient [32]. Smith et al. highlighted that formal training addressing communication with hearing-impaired older patients is highly underdeveloped [33]. In the context of the COVID-19 pandemic, there are even further barriers in communication. Not only distances in group interactions with older patients will become larger than 1 m, but personal protective equipment is a general sound barrier and prevents listeners from lip reading. After these external (patient independent) factors are optimally addressed, patient-centered interventions are only needed for specific subgroups of patients with advanced hearing impairment. 

A checklist of methods for clinicians to address hearing loss has been recently suggested [34]. Based on our findings of discharge planning meetings, we suggest that further study investigating real-life speech levels is needed to prove our finding. Further focus could then also focus on implementing interventions addressing health professionals. For example, specific voice and speach training for health professionals to attain clear speech with a minimum of 60 dBA could be implemented, and education of health professionals to systematically ask patients to repeat key aspects of the conversation to ensure a feedback loop. Furthermore, structural aspects should be reconsidered in an individual clinical context. For example, discharge planning meetings could be reorganized to a one-to-one setting instead of a group interaction, which might further facilitate communication with older inpatients. 

## 5. Conclusions

Our data show that real-life speech level differs between various types of group settings and suggest potentially inadequate speech levels by healthcare professionals. Further clinical studies investigating real-life speech levels by healthcare professionals are needed to support our findings. 

## Figures and Tables

**Table 1:** Descriptives and outcomes of speech levels.

	All	Discharge Planning Meetings	Memory Training Group	Chair Exercise Group
Demographics				
Recorded sessions [n]	31	21	5	5
Talk time, mean (SD) [min]	23.2 (8.3)	23.9 (6.6)	31.2 (5.0)	12.4 (6.3)
Attending persons, mean (SD) [n]	6.6 (1.4)	6.4 (1.2)	7.8 (0.8)	6.2 (1.9)
Proportion of talk time with speech level:				
<60 dBA ^(a)^, mean (SD) [%]	61.6 (32.0)	54.8 (32.5)	56.3 (25.4)	95.1 (4.6)
≥65 dBA, mean (SD) [%]	9.3 (14.3)	11.6 (15.3)	8.7 (15.3)	0 (0)
<55 dBA, mean (SD) [%]	17.1 (19.8)	11.4 (16.5)	17.2 (10.9)	40.6 (24.7)

Abbreviations: SD, standard deviation. ^(a)^ Speech level of <60 dBA corresponds to potentially inappropriate speech level according to definition (see Section 2.3).

## Data Availability

All data are provided in the present article.
